# Is *Porphyromonas gingivalis* involved in Parkinson’s disease?

**DOI:** 10.1007/s10096-020-03944-2

**Published:** 2020-06-21

**Authors:** Ingar Olsen, Douglas B. Kell, Etheresia Pretorius

**Affiliations:** 1 Department of Oral Biology, Faculty of Dentistry, University of Oslo, POB 1052 Blindern, 0316 Oslo, Norway; 2 Department of Biochemistry, Institute of Integrative Biology, Faculty of Health and Life Sciences, University of Liverpool, Liverpool, UK; 3 Department of Physiological Sciences, Faculty of Science, Stellenbosch University, Stellenbosch, South Africa

**Keywords:** Systemic inflammation, Cytokines, Hypercoagulation, Amyloid formation, Gingipains, LPS

## Abstract

*Porphyromonas gingivalis*, a major subgingival plaque bacterium in periodontitis, has recently attracted much attention as a possible microbial driver in Alzheimer’s disease. In the present paper, another common neuroinflammatory disease, Parkinson’s disease (PD), is discussed. A recent study found major virulence factors of *P. gingivalis* such as gingipain R1 (RgpA) and lipopolysaccharide in the blood circulation of a PD population. The current review reveals how features such as systemic inflammation, hypercoagulation, presence of amyloid fibrin(ogen) in plasma, and marked ultrastructural changes in platelets, probably induced by *P. gingivalis*, may affect the development of PD. Several other clinical studies have also demonstrated an association between periodontitis and PD. Even if the risk of periodontal diseases causing neurological disorders needs to be better substantiated, that should not keep us from trying to prevent them by performing careful daily dental hygiene.

## Introduction

Periodontitis is a collection of diseases where microorganisms cause destruction of the tooth-supporting structures through poorly controlled inflammatory responses. A world workshop held in 2017 distinguished periodontitis in three clinical forms: necrotizing periodontitis, periodontitis as a manifestation of systemic disease, and the forms of the disease previously recognized as chronic or aggressive, now grouped under a single category, periodontitis [1].

The oral cavity contains up to 1000 different bacteria localized in different niches, each with a characteristic microbiota [2]. A homeostatic balance exists most of the time between host and oral microbes, but this can be tipped towards disease. Traditional culture-based methods emphasized the anaerobic Gram-negative rods *Porphyromonas gingivalis*, *Tannerella forsythia*, and *Treponema denticola* (the red complex) as the major pathogens of periodontitis [3]. Culture-independent methods have extended this list to comprise both Gram-positive and Gram-negative bacteria (reviewed by Lamont et al. [4]). Among all these species *P. gingivalis* has emerged as a keystone bacterium in periodontitis [5, 6].

In periodontitis, polymicrobial communities initiate a dysregulated host response through polymicrobial synergy and dysbiosis [7]. As a keystone pathogen, *P. gingivalis* increases the nososymbiosity of subgingival microbial communities and drives periodontitis pathogenicity, remarkably, even at a low abundance [5]. In this interplay, inflammation is an important ecological factor that can stimulate outgrowth of periodontitis-associated microorganisms by tissue destruction releasing nutrients (e.g., degraded collagen, haeme-containing complexes, amino acids, and iron). The nutrients are transferred to the subgingival bacteria, to which *P. gingivalis* belongs, by the inflammatory exudate of the gingival crevicular fluid. Also increase in genes important for the pathogenesis of periodontitis such as proteolysis-related genes and genes for peptide transport and acquisition of iron and genes for synthesis of lipopolysaccharides (LPSs) have been detected. These genes elevate the proinflammatory potential of the microbial community [8]. Remodeling of the original biofilm into a dysbiotic one increases the capability of the biofilm to release proinflammatory cytokines from host cells [9]. *P. gingivalis* also uncouples inflammation from the bactericidal activity of leukocytes [5]. This subversive action disrupts the microbial homeostasis and contributes to development of a dysbiotic microbiota and periodontitis. Furthermore, *P. gingivalis* selectively suppresses IL-8 and T helper 1 cell-biasing chemokines (CXCL9, CXCL10, and CXCL11) [10]. By manipulating host immunity and keeping bactericidal and inflammatory activities apart, *P. gingivalis* increases the adaptive fitness of the entire microbial community [4].

## Neuroinflammation and Parkinson’s disease

**Fig. 1:**
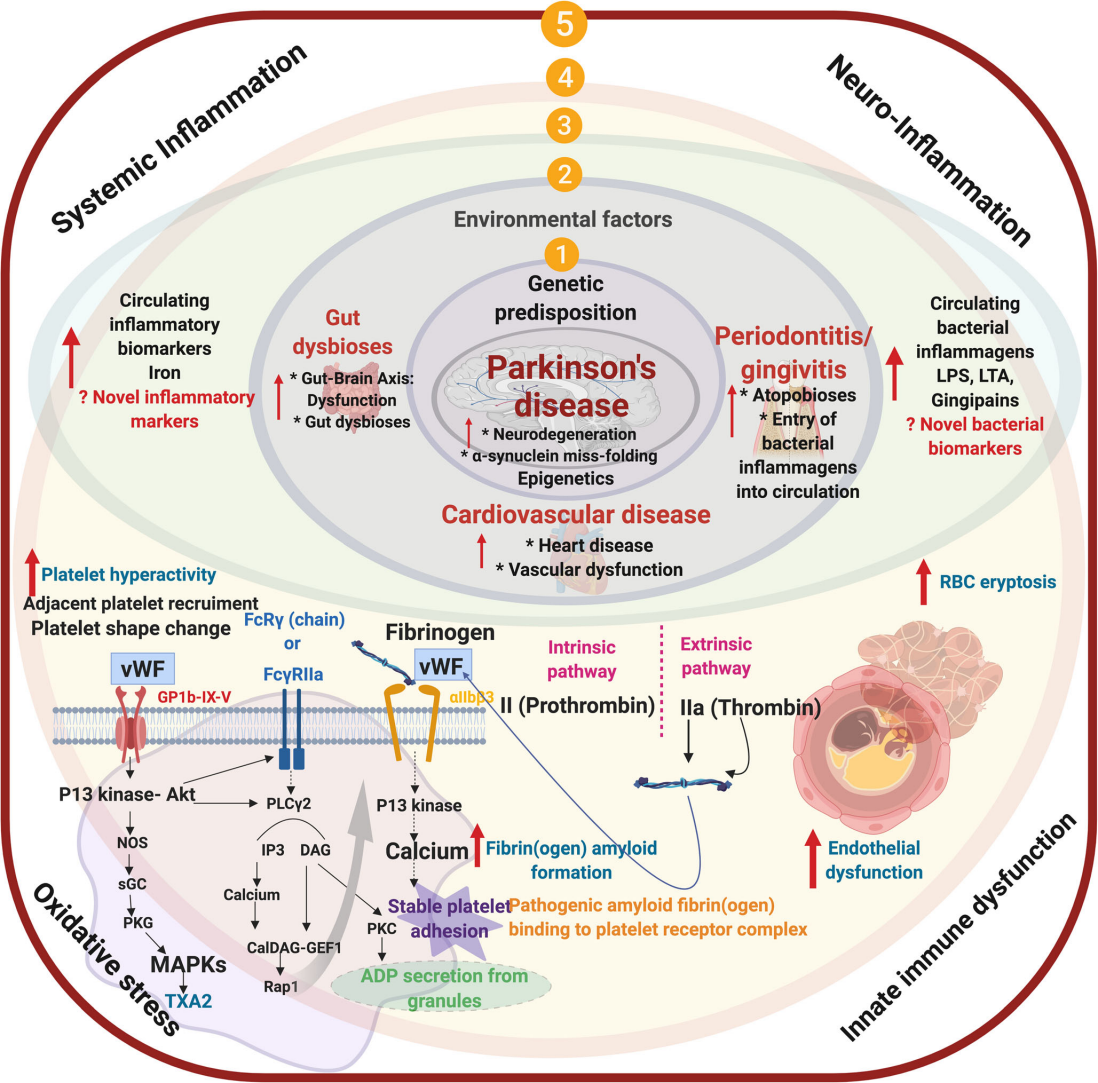
(1) Genetic/epigenetic predisposition and (2) environmental factors that culminate in an individual that will have gut dysbiosis, and/or gingivitis/periodontitis, vascular dysfunction; (3) an increased presence of circulating cytokines; resulting in dysregulated hematological system, e.g., **(**4) amyloid plasma proteins and increased propensity for hypercoagulation (an important hallmark of systemic inflammation), hyperactivated platelets; endothelial dysfunction; (5) ultimately resulting in Parkinson’s disease *also* being a true cardiovascular condition, where circulating inflammatory biomarkers (including bacterial inflammagens) may be used, not only as early detection of risk but also in tracking disease status

Parkinson’s disease (PD) is characterized by a number of pathologies (Fig. 1) from misfolding α-synuclein (αSyn) to neuroinflammation, mitochondrial dysfunction, neurotransmitter-driven changes in neuronal networks of the brain, and affection of the brain-gut axis (for a review, see Adams et al. [11]). The present review deals with the possible role of *P. gingivalis* as a driving force in developing PD, focusing on recent information.

Neuroinflammation is a characteristic feature of PD [12–14]. There are higher levels of inflammatory cytokines in the brain of PD patients than in controls, and inflammation seems to be an important factor in the process of neurodegeneration [15]. Furthermore, chronic inflammation, causing dysregulation of circulating inflammatory molecules, and an innate immune response seem to be important features in PD [16]. Both peripheral and brain inflammation contribute to initiation and progression of the neurodegeneration (for a review, see Adams et al. [11]). Increased levels of circulating cytokines such as IL-1β, IL-2, IL-10, IL-6, IL-4, TNF-α, C-reactive protein, RANTES (member of the interleukin-8 superfamily of cytokines), and interferon-gamma (INF-ɣ) have been found in PD [11, 17, 18], accompanied by oxidative stress [19]. In addition to significantly increased levels of circulating proinflammatory cytokines, PD was characterized by hypercoagulability (demonstrated by thromboelastograpy (TEG), confocal and electron microscopy) and an abnormal clotting potential [11]. Blood platelets showed marked ultrastructural changes and amyloid deposition was detected in the plasma [11].

## *Porphyromonas gingivalis* and Parkinson’s disease

**Fig. 2:**
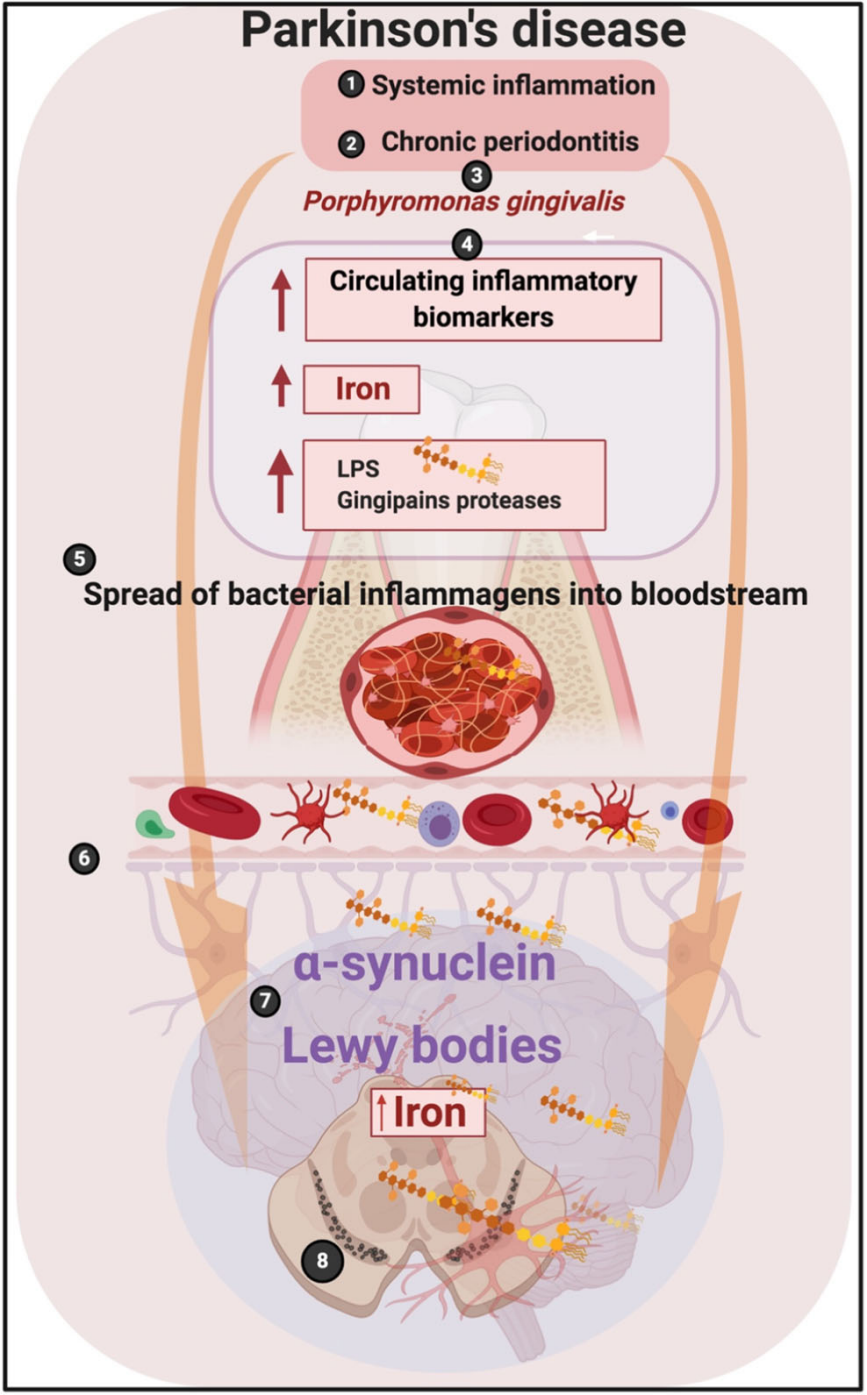
Many individuals with Parkinson’s disease suffer from both (1) systemic inflammation and (2) periodontitis. One of the bacteria that play a prominent role in the development of periodontitis is (3) *P. gingivalis*. (4) As a result of the systemic inflammation and periodontitis, there is an increase in circulating inflammatory biomarkers, including cytokines, iron, and bacterial inflammagens like LPS and proteases like gingipains. (5) The spread of these biomarkers via the bloodstream leads to a compromised blood-brain barrier (BBB) and an entry of particularly the bacterial inflammagens from *P. gingivalis* into the brain, where they may contribute to and fuel abnormal protein folding resulting in the formation of the abnormal presence of αSyn and Lewy body development, particularly in the dopaminergic neurons in the Parkinson’s disease brain

The reason for the chronic neuroinflammation in PD is unclear. Attention has been paid to dormant microbes, which can shed inflammagens such as LPS and lipoteichoic acid [20, 21]. These are ligands for Toll-like receptor 4 (TLR4) that can activate inflammation [22]. Bacterial inflammagens that recently attracted the attention in PD were proteases from the periodontal pathogen *P. gingivalis* [11] (Fig. 2). As mentioned, this bacterium is considered a keystone pathogen in periodontitis causing microbial dysbiosis typical of the pathogenesis. *P. gingivalis* may also be involved in other systemic inflammatory diseases such as type 2 diabetes mellitus, Alzheimer’s disease, rheumatoid arthritis, and cardiovascular disease (for reviews, see [11, 23]).

*P. gingivalis* has a number of ways to reach the brain from the infected periodontal pocket [24] and has been detected in the brain of animals and humans with Alzheimer’s disease [25, 26] where its LPS and cysteine proteases known as gingipains have been implicated in disease causation. As mentioned, proteolysis is important for this bacterium to obtain nutrition through breakdown of proteins. Gingipains are also important for degradation of antibacterial peptides [27] and for the bacterium to evade the complement system [28, 29].

## Hypercoagulation, proinflammatory cytokines, and plasma amyloid formation in Parkinson’s disease

In the study by Adams et al. [11], PD individuals were found to have a dysregulated profile of inflammatory biomarkers. Their whole blood was hypercoagulable with hyperactivated blood platelets and contained fibrin(ogen) with amyloid features. An inter-linked relationship between hypercoagulability, presence of inflammatory molecules, and activation of platelets was suggested. Furthermore, platelet pathology (hyperactivation, spreading, aggregation, or clumping), anomalous fibrin(ogen) protein structure, and red blood cell eryptosis were detected and interpreted as reflecting a systemic inflammation. The significantly upregulated cytokines (IL-1α, IL-1β, IL-17A, and TNF-α) detected were related to the change in the ultrastructure of hyperactivated platelets. Fibrinogen levels have also previously been found to be higher in PD patients than in healthy controls [30, 31]. In the study by Adams et al. [11], fibrinogen in clots polymerized into an increased number of β-sheets that reflected formation of an amyloid protein structure. These protein changes were visualized using fluorescent amyloid markers called Amytrackers. Such protein changes might affect the anomalous clot formation and emphasized the systemic nature of PD. Fibrin(ogen) that showed an amyloid protein structure (as shown by fluorescent amyloid markers including Amytrackers and thioflavin T) has also been detected in other diseases with systemic inflammation such as type 2 diabetes [32, 33] and Alzheimer’s disease [34] where it is a major feature.

## Gingipains and lipopolysaccharide in Parkinson’s disease

The gingipains Rgp and Kgp of *P. gingivalis* have been found to increase the thrombin time compared with controls [35]. Also other coagulation factors can be activated by gingipains such as factors IX and X and prothrombin [36, 37]. Therefore, the homeostatic control of the coagulation system/cascade can be disrupted when gingipains from *P. gingivalis* are present. Adams et al. [11] found, by using polyclonal antibodies, that gingipain R1 (RgpA) protease produced by *P. gingivalis* was present in platelet-poor plasma from PD patients. Furthermore, LPS from *P. gingivalis* caused hypercoagulability and RgpA hydrolyzed fibrin(ogen) so that healthy coagulation was inhibited. When RgpA and LPS were co-incubated, hyperclottable fibrin(ogen) could still be seen, supporting the findings that PD clots are dense and hyperclottable [38], and that a hyperclottable phenotype exists in PD patients. Whether the reducing effect of RgpA on clot formation, in terms of fibrinogen catalyzed by thrombin, also occurs in blood plasma in vivo, where protease inhibitors and other RgpA targets exist, is unknown. However, circulating bacterial inflammagens such as LPS have also previously been found in PD [39–42] where they could be involved in both development and progression of the disease. This implies that bacteria might participate in PD as drivers of the disease through endotoxins and exotoxins acting as potent inducers of cytokines [43].

Intestinal bacteria may also be involved in PD where the role of the gut-brain-microbiota axis has been emphasized [44–49]. However, this does not exclude involvement of oral bacteria since an oral-brain-microbiota axis may exist, as suggested recently for patients with autism spectrum disorder (ASD) [50]. Interestingly, intestinal dysbiosis has been associated with reduced LPS-binding protein in PD [51]. This protein may reverse the amyloid state of fibrin(ogen) [52]. Sampson et al. [53] reported that in mice, the gut microbiota was required for motor deficits, microglia activation, and αSyn pathology. Colonization of αSyn-overexpressing mice with microbiota from patients with PD increased physical impairments compared with microbiota transplants from healthy human donors.

## Other clinical studies supporting a potential periodontitis-Parkinson’s disease association

The study by Adams et al. [11] is not the only one reporting an association between periodontitis and PD. Chen et al. [54] found in a nationwide population-based case-control study that patients with periodontitis (*n* = 176) had a significantly higher risk of developing PD than controls (*n* = 275) with matching sex, age, index of year (occurrence of periodontitis), and comorbidity (adjusted hazard ratio = 1.431, 95% Cl [1.141–1.794], *p* = 0.002). In a similar study, Chen et al. [55] found that patients without periodontitis who underwent dental scaling over five consecutive years had a significantly lower risk of developing PD. Thus, dental scaling, which is the most common form of prophylaxis and treatment in periodontitis, significantly decreased the risk of developing PD. Other reports on an association between periodontitis and PD have come from Schwartz et al. [56], Zlotnik et al. [57], Kaur et al. [58], Hashioka et al. [59], and Olsen and Singhrao [60]. Although there are several studies indicating that periodontitis is more common in patients with PD, large longitudinal studies and randomized case-control or case-cohort studies are needed to substantiate this association [61].

## Concluding remarks

The keystone pathogen of periodontitis, *P. gingivalis*, has been used in this paper as an example of a possible bacterial involvement in PD. The finding of R1 (Rgpa) and LPS, major inflammagens of *P. gingivalis*, in the circulation of a PD population, supports a role for *P. gingivalis* in the development of PD. This has been supported by several clinical studies. *P. gingivalis* cells in brain tissue have not yet been detected, so the clarification of this question will have to await further research. Even if the risk of periodontal disease for the development of PD and other neurological disorders needs to be better substantiated, that should not keep us from trying to prevent them by performing periodontitis prophylaxis through careful daily cleaning of teeth.
